# Death of Professor Richardson

**Published:** 1845-10

**Authors:** 


### THE WESTERN JOURNAL.

New Series.—Vol. IV.—No. IV.

LOUISVILLE, OCTOBER 1, 1845.

### DEATH OF PROFESSOR RICHARDSON.

Died, on the 14th of September, at his residence near Lexington, Dr. William H. Richardson, Professor of Obstetrics and the Diseases of Women and Children in Transylvania University. The disease which terminated his life is said to have been typhoid fever, of which cases appear to have been occurring in Lexington and its neighborhood for two years past. The deceased, up to the time when he was seized with this fever, was in vigorous health, and, as he was of a longlived family, was looking forward to a green old age.

Dr. Richardson was a native of Kentucky, and of the county in which he spent nearly all the years of his active life. He studied medicine in Lexington, and afterwards attended medical lectures in the University of Pennsylvania. His primary education was much neglected, and he entered upon the practice of his profession in the spring of 1806 after hearing a single course of lectures. He settled in Georgetown, where he resided up to the commencement of the war in 1812. In the autumn of that year he went out as surgeon with one of the volunteer Kentucky Regiments after the downfall of Detroit; but was sent back with a sick officer. About this time he removed to Lexington, and in the year 1815 or 1816 was appointed Professor of Obstetrics in Transylvania University, then in a forming state. In 1816–'17 he delivered a few lectures on obstetrical medicine, but the University class was not organized until the fall of 1817; he then, in connexion with Drs. Blythe, Dudley, Drake, and Overton, delivered a full course. In the spring of 1818, after having been before the public as a teacher, he received the honorary degree of M. D. from the University of New York. In 1819 the medical department of Transylvania University was reorganized, and he resumed the duties of the chair which he continued to hold up to the time of his decease. He has the honor, therefore, of having been connected with the first scheme for medical teaching in the Valley of the Mississippi, and of those who were associated with him in the beginning he leaves but one behind him in the school. Of his earliest colleagues Dr. Dudley, Dr. Drake, and Dr. Overton survive him, but Dr. Dudley alone remains in Transylvania of the five professors who renewed the enterprise in 1819. Dr. Brown was the first to resign his place in the school, which he did in the spring of 1826, and the first also to pay the debt of nature, an event which took place not many years after his resignation. Dr. Blythe followed him in the order of his resignation and death, and Dr. Caldwell has been removed to a new theatre, where he is still zealously engaged in teaching. Dr. Richardson held on for more than a quarter of a century to the chair of his first choosing. Many resignations have occurred in that school from year to year; he never resigned. Dr. Drake gave up his professorship a second time, after four years' experience in the school, and subsequently four out of the six members of the Faculty came to the Medical Institute of Louisville; but Dr. Richardson, while he was sensible of the superior advantages which this school would enjoy, made up his mind to continue in the institution which he had been instrumental in building up. Amid all the changes which have since taken place in Transylvania, he remained fixed where started.

The wonder has often been expressed, that a teacher so deficient in everything like scholarship should have been able to maintain so respectable a rank for so many years. Professor Richardson's education unquestionably was very limited. He was totally ignorant of every language except our own, and even of that he possessed but an imperfect knowledge, insomuch that he had a repugnance to writing, and hence has left behind him in a durable form but little of his experience acquired during nearly forty years of diligent practice.

It cannot be denied that this destitution of preliminary learning detracted from his dignity and usefulness as a teacher, for young men, struck with his grammatical blunders, were too prone to conclude that he must also be wanting in professional knowledge. It was the impression of his first classes that he appropriated not only the thoughts but the language of Burns too extensively in his lectures; but his judgment and ability as an obstetrical practitioner were never questioned, and by much reading it is certain that he made amends, in subsequent life, for the imperfections of his early teaching. He never became a scholar; but he corrected, one after another, the more glaring faults which provoked the merriment of his first pupils, and, what was of still higher consequence, kept his mind informed of all the improvements in his branch of the profession. As he never aspired to originality, and had no cherished theory to defend, his understanding was ever open to conviction, and he was prepared to welcome knowledge from whatever quarter it might approach him.

If we were asked upon what did his success as a teacher depend, we should answer, mainly upon his social qualities. These attached strongly to him a large body of his pupils, and made him a favorite in the community of which he was an active member. Students, who felt a warm personal regard towards him, could make liberal allowances for the absence of literary grace in his lectures. But to many amiable social qualities, and many manly traits of character, he added, also, a style of lecturing which pleases the ear of Western young men. His elocution, indeed, with the finish which a correct knowledge of his native language would have imparted to it, would have been highly pleasing, and even impressive. His manner of speaking was loud, earnest, and oftentimes vehement, after the fashion of the stump. He was of a bold, impulsive temperament, and his confident air, aided by an agreeable voice, fixed the attention of the less fastidious portion of his audience. When to all this we add, that he was experienced as an obstetrician, and was reputed to possess peculiar skill in that department, we have, perhaps, the elements which contributed chiefly to maintain him, in spite of his acknowledged deficiencies, for so long a time in his elevated position before society. Undoubtedly, he was much better versed in medical science than in letters, and in our profession, we know, that instances are not wanting, of men attaining to the highest rank with the slenderest literary acquisitions. John Hunter would have made an exceedingly sorry figure among scholars, but, nevertheless, he made himself the first surgeon of his age.

Professor Richardson was greatly beloved by his private pupils, and his memory will be held in grateful remembrance by many who shared in his personal kindness. Of those whom he instructed some are now teachers themselves, from among whom it would seem meet that his successor should be chosen.

This hasty notice has been written without the materials necessary for a biographical sketch of the deceased, but some friend familiar with his history, we hope, will favor the profession with an extended memoir of his life and character.

We are happy to be able to add, that Professor Richardson was many years a member of the Baptist church, and that he was calm and resigned in view of his approaching dissolution.

